# Impact of the Degree of Maturity of Walnuts (*Juglans regia* L.) and Their Variety on the Antioxidant Potential and the Content of Tocopherols and Polyphenols

**DOI:** 10.3390/molecules24162936

**Published:** 2019-08-13

**Authors:** Karolina Pycia, Ireneusz Kapusta, Grażyna Jaworska

**Affiliations:** Department of Food Technology and Human Nutrition, Faculty of Biology and Agriculture, University of Rzeszow, Zelwerowicza 4 St., 35-601 Rzeszow, Poland

**Keywords:** walnut, maturity, antioxidant properties, polyphenols, tocopherols

## Abstract

The aim of the study was to characterize the antioxidant properties; establish the profile of polyphenolic compounds and evaluate the content of tocopherols in walnuts of three varieties (Leopold; Apollo; Resovia) differing in the degree of maturity (harvest date). The profile of polyphenolic compounds was established by UPLC-PDA-ESI-MS. The content of tocopherols was determined by HPLC-FLD. It was found that the content of dry matter and fat increased and the antioxidant properties decreased with the maturation of nuts. Walnuts of the Leopold cultivar harvested in July exhibited the highest content of total polyphenol (2149.08 mg/100 g dry mass). In their polyphenolic profile; 26 compounds were identified; mainly belonging to the class of ellagitannins. The polyphenolic content decreased with the maturation of nuts. The total content of tocopherols in the tested nuts increased with ripening and ranged from 1.76 mg/100g (Apollo VII) to 18.30 mg/100g (Resovia IX)

## 1. Introduction

In recent years, nuts and their health-promoting properties have generated great interest among scientists. The results of clinical and epidemiological studies clearly indicate that regular consumption of nuts, mainly walnuts, reduces the risk of cardiovascular disease, diabetes, cancer and inflammatory diseases [1]. According to many authors [2,3,4], these properties result from the chemical composition of walnuts. First of all, they are a rich source of fat (up to 70% dry mass), including unsaturated fatty acids, as well as phytochemical compounds such as phenols, tocopherols and sterols. In addition, walnut kernels contain polyunsaturated fatty acids (PUFAs) that lower LDL cholesterol and raise HDL cholesterol [5]. Moreover, walnuts contain dietary fiber and essential micronutrients [4]. Nuts are also a rich source of complete proteins, which are abundant in exogenous amino acids as well as minerals, including potassium and magnesium [6]. In walnut oil, there are a number of active phytochemical compounds which predominantly consist of polyphenols [7]. In terms of the content of polyphenols, walnuts dominate among all nuts; their average polyphenol content is about 1591.5 mg/100 g, and in hazelnuts it varies within wide limits from 291 mg/100 g to 875 mg/100 g [8]. Of all polyphenols identified in walnuts, ellagitannins constitute the most numerous group [9], followed by phenolic acids, flavanols and dihydrochalcones [10,11].

Walnuts (*Juglans regia* L.) belong to the *Juglandaceae* family. The native region from which they originate is Central Asia, the western Himalayan chain, the region of Kyrgyzstan. They reached Europe before Roman times, and later spread to America and northern Africa [4]. Walnuts and hazelnuts (*Corylus avellana* L.) constitute about 60% of nut production in Europe. On the global scale, the US and China are the major walnut producers [11]. Recently, there has been a significant increase in nut consumption around the world. Probably, this trend is due to the numerous health benefits of nuts and nutritional programs that promote their consumption. In general, the nuts are consumed raw, roasted, salted or in the form of a snack. For most consumers, the kernels are the edible parts of the nuts. Their rich chemical composition and related pro-health properties are well documented [6,10,12]. According to numerous reports, biologically active compounds may also be found in other non-edible parts of the fruit, including in co-products, such as skin, endocarp or exocarp [10,13,14,15]. During the maturation of the fruit, the profile and polyphenol content change significantly. Fruits, after reaching consumer maturity, contain smaller amounts of polyphenols, but more natural dyes (carotenoids, anthocyanins) in the skin [11,16]. The ripening process of walnuts and hazelnuts differs significantly from other fruits. In the case of walnuts, edible seeds are surrounded by a hard, woody shell, which in juvenile fruits is covered with an additional green layer, very rich in polyphenols [11,14]. The kernels are in the form of two cotyledons joined together, covered with a thin membrane, which accounts for 5% to 10% of the seed weight [10,11]. It has an important protective role, as it prevents the oxidation of fat. According to established research, the vast majority of antioxidants are found in the peel of nuts or in the green husk surrounding the fruit, and are lost during ripening. In addition, the processing of nuts preceding their use in the bakery or confectionery industry (roasting, blanching) significantly reduces their nutritional value [10,14,16]. Thus, consumption of unprocessed, natural, unripe nuts may be valuable in terms of pro-health properties in the case of most nuts, but almonds and pistachios should be consumed roasted [17].

Until now, researchers have generally focused on the analysis of the profile and content of bioactive substances in fully mature, dry nut kernels. Meanwhile, the green husk of nuts appearing in immature fruits and therefore the unripe nuts are also valuable in terms of pro-health properties. The green husk protects the fruit from harmful UV radiation and the attack of herbivores. On the other hand, it is a very rich source of strong antioxidants with pro-health properties. The literature on the subject lacks data on the influence of the walnut variety and the term harvest (degree of maturity) on antioxidant properties and the content of biologically active compounds in whole walnut fruits. Therefore, the aim of the study was to assess the impact of the degree of maturity of different varieties of walnut fruits cultivated in Poland on antioxidant properties and the content and profile of selected biologically active substances.

## 2. Materials and Methods

### 2.1. Materials

#### Samples

The research material consisted of three cultivars of walnut fruits (*Juglans regia* L.) (Resovia, Leopold, Apollo). Walnut fruits were harvested in 2017 in the Świętokrzyskie Province (Zbigniewice Kolonia) (50°37’00”N, 21°30′16”E, Poland). Samples of walnuts weighing 1 kg each were collected during their maturation, at equal intervals of 31 days (July 1, August 1 and September 1). After the harvest, walnuts were cut into pieces. From the nuts collected in September, the woody shell was removed because it was difficult to grind the lyophilized nuts. Walnuts were dried in a lyophilizer (Alpha 1-2 LDplus, Martin Christ GmbH, Osterode am Harz, Germany) at −45 °C to constant weight, then ground and stored at −18 °C until analysis.

#### Reagents and Standards

The reagents used for the antioxidants analysis (ABTS, DPPH, FRAP methods) and Folin–Ciocalteus reagent were purchased from Sigma-Aldrich (Poznań, Wielkopolska, Poland). Methanol purchased from Chempur (Piekary Ślaśkie, Silesia, Poland), were used in the phenolic extraction procedure. The oil ether for the extraction of fat by the Soxhlet method came from Chempur. The following standards were used for quantification of phenolic compounds: P-coumaric acid, ellagic acid, agrimoniin, procyanidin B, gallic acid were from Extrasynthese (Genay, Lyon, France). The chemicals for the mobile phases were the HPLC-MS grade acetonitrile and formic acid from Fluka Chemie (VWR International, Gdańsk, Pomerania, Poland). Water for the mobile phase was double distilled and purified with the Milli-Q system (Millipore, Bedford, MA, USA). The standards α, β, γ, δ -tocopherol were purchased from Sigma (Poznań, Wielkopolska, Poland). 

### 2.2. Methods

#### 2.2.1. Analysis of Physicochemical Properties

In the analyzed nuts, dry matter content was determined prior to lyophilization by means of a reference method [18,19]. In turn, in dried nuts, the content of fat was determined by Soxhlet extractions and the mineral content by burning the sample in a muffle furnace at 950 °C [18]. The analyses were performed in triplicate.

#### 2.2.2. Analysis of Antioxidant Properties

For the analysis of antioxidant potential and total polyphenols, methanol extracts of walnuts were used. A sample of lyophilized walnut (5 g) was treated with a 70% methanol solution (20 mL). The extraction process (30 min at 25 °C) was carried out using an ultrasonic bath (Sonic 10, Polsonic, Poland). The one-stage extraction was used. After its completion, the samples were centrifuged for 10 min at 7000 rpm. The clear methanol extract was used for further analysis.

The antioxidant activity was carried out using the ABTS^+^ cation radical by a spectrophotometric method according to Re et al. [20]. The extracts of the sample were reacted with the ABTS reagent in the water solution. The reaction mixture consisted of adding 0.03 mL of the sample and 3 mL of the ABTS radical solution in the water. The absorbance at 734 nm was measured after 6 min of reaction. The blank test was distilled water. The scavenging activity was measured according to the elimination of DPPH (1,1-diphenyl-2-picrylhydrazyl) free radicals [21]. The extracts of the sample were reacted with the stable DPPH radical in the methanol solution. The reaction mixture consisted of adding 0.5 mL of the sample and 2 mL of the DPPH radical solution in methanol. The changes in color (from deep violet to light yellow) were read (Abs) at 517 nm after 10 min of reaction. The blank test was methanol. The Ferric reducing antioxidant power (FRAP) was determined according to Benzie and Strain [22]. The extracts of the sample were reacted with the stable FRAP reagent. The reaction mixture consisted of adding 0.5 mL of the sample and 3 mL of the FRAP reagent. The blank test was distilled water. The absorbance was recorded at a wavelength of λ = 593 nm. Determination of ABTS, DPPH and FRAP was performed using a spectrophotometer (Nicolet Evolution 300, Thermo, Waltham, MA, USA). The concentration range of Trolox on the calibration curve was 0–100 nmol Trolox/0.5 mL. All analyses were carried out in triplicate. The values of the antioxidant activity of walnut extracts (ABTS, DPPH, and FRAP) were expressed in mM TE/100 g dry matter (Trolox Equivalent).

The total phenolic content was assessed using the Folin-Ciocalteu phenol reagent at a wavelength of 765 nm [23]. The reaction mixture contained 0.1 mL extract, 2 mL distilled water, 0.2 mL Folin-Ciocalteu reagent and 2 mL sodium carbonate solution (20 g/100 g). The blank test was distilled water. The total phenolic content was expressed as gallic acid equivalents (GAE) in milligrams per gram dry mass of walnut fruits (GAE; mg/100 g dry matter). The analysis was carried out in triplicate.

#### 2.2.3. Analysis of Profile of Polyphenolic Compounds

The analysis of polyphenolic compounds profile was carried out in walnut fruits of all three varieties and in all three stages of ripeness. Qualitative and quantitative analyses of polyphenols were performed using the UPLC Waters Acquity system (Waters, Micromass, Manchester, UK) consisting of a photodiode array detector (PDA) and tandem quadrupole mass detector (TQD) with electrospray ionization, according to previous investigation [19,24]. The ion source operating parameters were as follows: Cone voltage 45 V, capillary voltage 3.5 kV, extractor 3 V, RF lens 100 mV, source temperature 120 °C, desolvation temperature 350 °C, desolvation gas flow 800 L/h, cone gas flow 100. The collision cell parameters for the MS/MS experiments were as follows: gas collision flow (argon), 0.3 mL/min, and collision energy 30 eV or 40 eV. Acquisition in the MS scan and daughter scan was performed in the negative ion mode. Polyphenolic compounds were separated on a 100 mm × 2.1 mm i.d., 1.7 μm Acquity BEH column (Waters) using a linear 8.5 min gradient from 5% to 100% of solvent B (40% acetonitrile containing 0.1% formic acid) in solvent A (water containing 0.1% formic acid) with a flow of 0.35 mL/ min. The injection volume of the samples was 5 μL. Analyses were carried out in triplicate. The runs of polyphenolic compounds were monitored at the following wavelengths: Ellagitannins at 240 nm, hydroxycinnamates at 320 nm. The characterization of the single components was carried out via retention time (Rt), spectra, accurate molecular masses, literature data and pure standards, if available. Calibration curves at concentrations ranging from 0.05 mg/mL to 5 mg/mL (*r^2^* ≤ 0.9998) were made for the p-coumaric acid, ellagic acid, agrimoniin, procyanidin B, and gallic acid as standards.

#### 2.2.4. Analysis of Tocopherols

To analyze the content of tocopherols, oil extracted from walnuts using the Soxhlet method was used [18]. Tocopherol contents (α, β, γ, δ) were measured by the high-performance liquid chromatography (HPLC) according to the method by Pycia et al. [19]. The chromatography system consisted of a SYKAM chromatograph (Eresing, Landsberg am Lech, Germany), using a 5C18-MS-II column (200 mm × 4.6 mm i.d., 5µm Cosmosil), 100% methanol as a mobile phase at a flow rate of 0.8 mL/min and a fluorescence detector (Shimadzu RF 353, Kyoto, Japan)) with the excitation wavelength set at 290 nm and the emission wavelength at 330 nm. The injection volume of a measured sample was 20 µL. Tocopherols were quantified using standard curves calculated by the linear regression analysis. Analyses were carried out in triplicate.

#### 2.2.5. Statistical Analysis

Statistical analysis was carried out using Statistica version 12.0 (Stat-Soft, Krakow, Poland). For differences in analyzed parameters among individual sampling data, two-way analysis of variance (ANOVA) was calculated and the Duncan test was used to distinguish among results (*p* ≤ 0.05). the coefficients of linear Pearson’s correlation between selected physicochemical and antioxidant parameters were computed. Reported values of correlation coefficients are significant at the level *p* ≤ 0.05. Additionally, the principal component analysis (PCA) was used to provide a ready means of visualizing the differences and similarities among the investigated walnut cultivars in different degrees of maturity.

## 3. Results and Discussion

### 3.1. Physicochemical Properties

Table 1 compares the values of basic chemical parameters characterizing walnuts of different cultivars, harvested at three time periods. A two-factor analysis of variance showed a significant impact of the time of harvesting, their varieties and the interaction of both factors on the dry matter content (*p* < 0.001). It was found that the average content of the dry substance of the tested nuts increased with the ripening of fruits from 22.88% (July) to 64.84% (September). However, among the nuts of the analyzed varieties, the highest content of dry matter was found in the nuts of the Resovia cultivar collected in September. The increase in the content of dry substance in nuts probably results from the formation of the nucleus of the fruit. The ripening process causes the dehydration of nuts. This is in accordance with previous observations of other authors. Ballistreri et al. [25] reported that unripe and ripe pistachio nuts had a water content of 50.7% and 35.3% respectively, and nuts subjected to drying only 3.3%. The fat content is the decisive factor for the energy value of nuts. A two-factor analysis of variance demonstrated a significant impact of the degree of nut maturity, the variety and the interaction of both factors on the value of this parameter (*p* < 0.001). It was found that the content of the nutrient increased as ripening increased.

The average fat content in nuts collected in September was 22.64 g/100 g d.m. and it was more than three times higher than the average fat content in nuts obtained in July (6.00 g/100 g d.m.). The highest fat content was detected in the kernels of Apollo and Resovia cultivars collected in September. The fat content of nuts collected in September is about three times lower as compared to the fat content of the dry nut seeds, which according to different authors ranges from 63.0% [26] to 67.4% [27]. A two-factor analysis of variance demonstrated that both the harvest date and the walnut variety had a significant effect on the mineral content, while the interaction of the two factors was negligible (*p* = 0.123). The content of total mineral components in the investigated nuts decreased as they ripened. The highest average ash content was recorded for walnuts harvested in July (5.94 mg/100 g d.m.), and the smallest for nuts collected in September (3.81 mg/100 g d.m.). The literature lacks information on the impact of the degree of maturity of walnuts on the content of minerals. Researchers disagree about the value of this parameter in dry walnut kernels; according to Amaral et al. [28], the average mineral content is around 2%. Pereira et al. [12] indicate a higher value of 4%. Korsteiner et al. [29] claims that differences in the chemical composition of walnuts result from genetic differences, growing conditions, the natural environment, chemical composition of soil, stage of maturity as well as storage conditions.

### 3.2. Antioxidant Properties and Polyphenol Content

Table 2 illustrates the antioxidant activity determined by the ABTS, DPPH, FRAP assays and the average total polyphenol content in unripe walnuts of different varieties.

The two-factor analysis of variance showed a significant effect of the degree of maturity (harvest date), nut varieties and mutual interaction of these factors (*p* < 0.001) on the values illustrating the antioxidant capacity of nuts and the content of their polyphenolic compounds. It was found that the antioxidant potential of nuts decreased significantly with their maturation, i.e., the later date of harvesting. Probably the reason for this is the presence in green, i.e., immature walnuts, of green scales, which is an excellent, natural source of polyphenolic compounds with very strong antioxidant properties. This is confirmed by research carried out by Oliveira et al. [14]. The researchers reported that the average total polyphenol content in the green walnut shells was dependent on the variety and was around 74.08 mg GAE/g, which confirms the very strong antioxidant properties of immature nuts. This is confirmed by the research of other authors [28,30]. Stampar et al. [30] analyzing the polyphenol profile of the green walnut extract, indicated about 13 compounds with a high antioxidant capacity, mainly belonging to phenolic acids and catechins. In addition, Pereira et al. [12] reported strong antioxidant properties not only of immature nuts, but also of walnut leaves. These authors in the spectrum of polyphenols extracts from walnut leaves identified as many as 10 polyphenols. In our study, nuts harvested in July had the highest mean value of antioxidant potential expressed by the ability to reduce ABTS+ cation-radical, DPPH and FRAP radical (49.04 mmol Trolox/100 g d.m., 49.83 mmol Trolox/100 g d.m., 96.75 mmol Trolox/100 g d.m.). On the other hand, with maturation, in September, a significant decrease of antioxidative potential was observed on average by 85% (ABTS +), 36% (DPPH) and 25% (FRAP) as compared to samples obtained in July. Among the analyzed cultivars, the highest antioxidant capacity was found in the Leopold nuts harvested in July (Table 2), while the smallest was found in the Resovia nuts obtained in September. The total polyphenol content determined by the Folin-Ciocalteu method varied within wide limits from 2149.08 mg GAE/100 g d.m. (Leopold, July) to 498.12 mg GAE/100 g d.m. (Resovia, September). Therefore, the value of the discussed parameter decreased with the harvesting time in the following sequence: July > August > September (Table 2). It was found that the total polyphenol content was strongly positively correlated with the antioxidant activity measured by the method of ABTS and DPPH (respectively *r* = 0.81, *r* = 0.83, *p* < 0.01). During ripening, the structure of the nut changes; the green husk, which is very rich in polyphenols, turns into a hard shell, which is inedible. For this reason, unripe walnuts contain more polyphenols [11,30]. The literature on the subject is mainly dominated by data on the total polyphenol content in dry kernels. Researchers report different ranges of the value of the discussed parameter. Pycia et al. [19] claim that the total polyphenol content in 11 analyzed varieties of walnut varied from 0.82 g GAE/100 g to 2.09 g GAE/100 g d.m. and it was statistically significantly dependent on the variety of nuts. The above values are higher than those from the literature [31]. According to the authors cited, the average total polyphenol content in walnuts is about 1591.5 mg/100g. On the other hand, Cerit et al. [32] reported a much higher total polyphenol content (TPC) value in nuts cultivated in Turkey. The authors stated that the content of polyphenols in walnuts ranged from 34.0 mg GAE/g to 50.3 mg GAE/g. Thus, the quoted values are 2–3 times higher than other literature data. Such large variation among authors regarding the total polyphenol content in walnuts may result from factors before and after collective nuts. The most important factors are the climatic and soil conditions, the variety and its modifications, the conditions for harvesting and storage of seeds and the conditions for extraction of the raw material [32].

### 3.3. Profile and Content of Phenolics

Table 3 summarizes the profile of polyphenolic compounds of the tested walnuts, determined by the UPLC-PDA-MS/MS analysis. On the basis of spectral characteristics (UV-Vis and collision induced dissociation—CID), 26 compounds were identified. Major phenolic compounds found in methanolic extracts of walnut cultivars belonged to the class of ellagitannins. The characteristic feature of these compounds was the common MS/MS ion observed at *m*/*z* 301. This diagnostic ion corresponded to the ellagic acid derived from the hexahydroxydiphenoyl group (HHDP) relesed after CID. These compounds are characterized by their hexahydroxydiphenoyl (HHDP) group, which is released on acid hydrolysis and spontaneously lactonizes to the ellagic acid. Ellagitannins represent a large group of polyphenolic compounds which has been extensively studied and which, as well as the ellagic acid itself, has recently attracted considerable attention due to their high antioxidant activity [33]. Additionally, the ellagic acid has recently gained increasing interest due to beneficial properties and the health benefits associated with the intake of foods containing high ellagitannins levels in the prevention of cardiovascular diseases [24] as well as neoplastic diseases [34,35].

Most of the identified ellagitannins occurred in several isomeric forms. The most abundant ions were observed for five isomeric compounds with (M − H)^−^ at *m*/*z* 935 (peaks 8, 9, 13, 14 and 16), yielding the main fragment ions at *m*/*z* 301, which corresponds to the ellagic acid (M − H− 481, loss of HHDP-glucose) and at *m*/*z* 481, which corresponds to the deprotonated HHDP-glucose (M – H − 302, loss of HHDP). This fragmentation pattern corresponds to a bis-HHDP-glucose structure, presumably pedunculagin or casuariin isomers, which have been previously reported as among the major ellagitannins in walnuts [36].

The second most abundant recurring compound was the glansrin C isomer, for which three repetitive peaks were observed at *m*/*z* 933 (peaks 10, 12 and 22) and fragment ions: *m*/*z* 631 (loss of HHDP), *m*/*z* 481 (M − 452, loss of the trigalloyl group) and *m*/*z* 301. The presence of glansrin C in walnuts has also been confirmed in earlier studies [33].

The remaining identified ellagitannins showed a similar fragmentation pattern to already identified compounds [27,37]. In the analyzed nuts a larger number of polyphenolic compounds was identified as compared to the few data found in the existing literature. In the present study more polyphenol compounds were identified compared to Persic et al. [11]. According to the authors’ analysis, the spectrum of polyphenols of walnut and hazelnut seed was characterized by only sixteen polyphenols, among which derivatives of the hydroxycinnamic acid, hydroxybenzoic acid and flavonoids derivatives were determined. The ellagic acid and quinic acid were detected. In this study, whole nuts were analyzed, i.e., a seed with a cover, hence probably the identification of a larger number of polyphenols. Further, Persic et al. [11] reported that in the nuts of three analyzed cultivars (RW, Lara, Fernor) the venillic acid hexoside dominated among the phenols, and its content increased during maturation.

It was also found that the dominant group of polyphenols in the analyzed nuts was ellagitannins. Regueiro et al. [27] demonstrated that the peel surrounding the kernel is rich in hydroxybenzoic acid derivatives, particularly in ellagitannin. Persic et al. [11] reported that in walnuts there were also catechins, which were the major compounds in the flavonoid group. The content of these compounds was the highest in the immature nuts, and it decreased during ripening.

Table 4 presents the quantitative composition of the identified polyphenols in the examined walnuts of different cultivars, differing in the degree of maturity. The total polyphenol content determined by the UPLC-PDA-MS/MS method ranged from 114.77 μg/g (Resovia, September) to 543.10 μg/g (Leopold, July). In general, the content of polyphenols in walnuts of different varieties decreased with the maturity of the fruit. Among the nuts harvested in July, the highest content of polyphenols was observed for the Leopold cultivar, and the lowest for Resovia. In addition, the largest decrease in the content of the compounds during ripening was observed for the Leopold variety. The content of polyphenols in nuts obtained in September was 80% lower compared to nuts collected in July. In the polyphenol profile, in terms of quantities, the praecoxin A/platycariin isomer (trigalloyl-HHDP-glucose) dominated for all tested samples (Table 4). The walnuts of all varieties harvested in July were characterized by the highest content of this derivative. Furthermore, the content of the quinic acid and gallic acid in Leopold walnuts decreased as it matured. A similar situation was observed for the ellagic hexoside. It was found that in the nuts of Leopold and Resovia cultivars, the content of the discussed compound decreased with extended harvesting time, in the following order: July > August > September. The dynamics of the changes in the content of polyphenols and dicarboxylic acids was also analyzed by Persic et al. [11]. They observed that in individual varieties of nuts, the content of hydroxybenzoic acid derivatives, flavonoids and dicarboxylic acid derivatives decreased in general as nuts ripened.

### 3.4. Profile and Content of Tocopherol

Table 5 shows the composition of tocopherols in the investigated walnuts. Four forms of tocopherol were identified: α, β, δ and γ. In terms of quantity, the sum of β and δ of tocopherols was determined. The two-factor analysis of variance showed a significant impact of the harvest time, the variety of nuts and the interaction of the two factors on the content of tocopherols. The content of these compounds increased as the walnuts matured, because their average content in nuts harvested in September was about three times higher than the samples obtained in July. This is probably due to the higher fat content in mature nuts (September) (Table 1). This is also confirmed by the statistical analysis, which showed a strong positive linear correlation between the fat content in nuts and the content of α and γ derivatives and the sum of β and δ tocopherols (respectively *r* = 0.68, *r* = 0.72, *r* = 0.88, *p* < 0.01). Analyzing the levels of individual fractions, it was shown that the average content of α-tocopherol in the oil from nuts collected in July was 1.18 mg/100 g d.m., γ-tocopherols 1.98 mg/ 100 g d.m. and the sum of β and δ of tocopherols 0.44 mg/ 100 g d.m. In the Apollo nut oil only the γ form of tocopherol was found. In contrast, the Resovia oil obtained in July was dominated by the very valuable in terms of nutrition, α form of tocopherol. On the other hand, in nuts harvested in August, the average content of tocopherols was 8.63 mg/100g d.m. and it was 139% higher compared to nuts from July. Among the tested varieties, the walnuts of the Resovia cultivar were characterized by the highest content of the discussed group of compounds. In the tocopherol profile of this nut variety, the sum of β and δ of tocopherols predominated (Table 5). Moreover, the Resovia nuts also had the highest content of tocopherols among nuts harvested in September. The smallest average value of the discussed parameter was present in Leopold cultivar nuts. The statistical analysis showed a strong positive linear correlation between the content of individual tocopherol fractions. It was found that the content of the α-tocopherol fraction correlated positively with the content of beta tocopherols and the sum of beta and delta tocopherols (respectively *r* = 0.57, *r* = 0.89, *p* < 0.01). The literature on the subject lacks data on the impact of the degree of maturity of walnuts on the content of tocopherols. The only available information is about the value of the discussed parameter in dry kernels of various nuts. Thus, the mean, total levels of tocopherols determined in the study are lower than those given by other authors. Korsteiner et al. [29] reported that the content of these substances in the walnut dry kernel oil was 25.7 mg/100 g, with no α-tocopherol fraction identified. Higher levels of tocopherols in the walnut oil were also observed by Abdellah et al. [6] and Pycia et al. [19]. The authors demonstrated that the content of tocopherols in the analyzed cultivars of walnut varied statistically significantly from 186.54 mg/kg (Local gd) to 283.11 mg/kg (Parisienne) and from 19.83 mg/kg (U17) to 126.00 mg/kg (Lake) respectively. In addition, Kornsteiner et al. [29] noted a relationship between the nut species and the α-tocopherol content. They found that the level of this fraction decreased with the type of nuts in the following order: Hazelnuts (*Corylus avellana*) > almonds (*Prunus dulcis*) > peanuts (*Arachis hypogaea* L.) > pistachios (*Pistacia vera*) > pine nuts (*P. pinea*) > walnuts (*Juglans regia* L.) > Brazil nuts (*Bertholletia excelsa*) > pecans (*Carya illinoinensis*) > cashews (*Anacardium occidentale* L.) > macadamia nuts (*Macadamia ternifolia*). Maguire et al. [38] stated that the α-tocopherol fraction dominated in the profile of almonds, peanuts, hazelnuts and macadamia.

Tocopherols and tocotrienols occur in four forms: α, β, δ and γ. These forms differ in biological activity. They exhibit strong antioxidant properties [39]. According to Abdellah et al. [6] and Li et al. [40], the form of γ-tocopherol is more effective compared to the form of α-tocopherol in the prevention of platelet aggregation leading to atherosclerosis or in the prevention of oxidation of the LDL cholesterol fraction.

### 3.5. Principal Component Analysis

**Figure 1:**
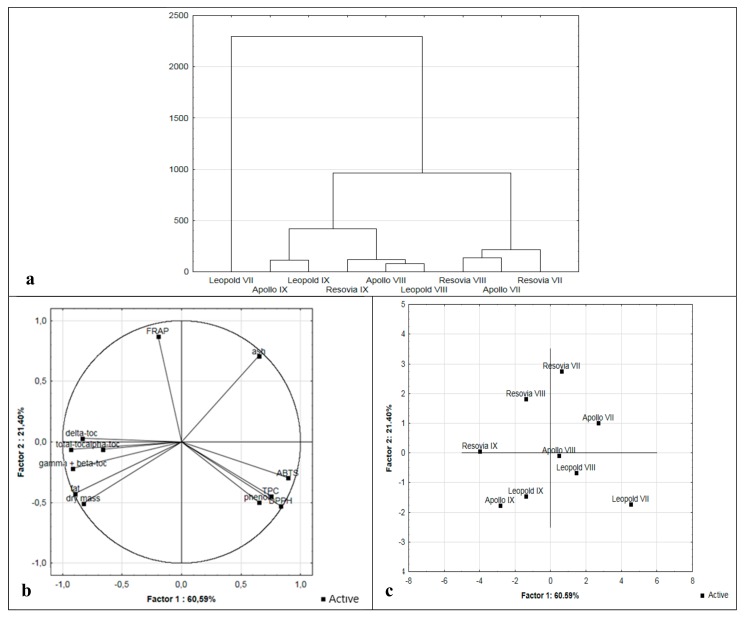
Analysis of main components: (**a**) Dendrogram; (**b**) distribution of the analyzed parameters; (**c**) distribution of walnut samples.

The obtained data were analyzed statistically by means of the cluster analysis and principal component analysis (PCA). A dendrogram was obtained as a result of the Ward grouping (Figure 1a). Analysis of the obtained clusters allowed three groups of the analyzed walnut samples to be distinguished. The first group (Apollo IX, Leopold IX) contained the samples with the highest content of dry matter and fat, a high content of individual tocopherols and the highest value of antioxidant potential among samples of nuts obtained in September. The second cluster consisted of the Leopold and Apollo cultivars harvested during August and the Resovia variety harvested in September (Resovia IX). The third group of samples comprised nuts obtained in July (Resovia, Apollo), which were characterized by the lowest content of dry matter and fat, but the highest antioxidant potential. In addition, this group includes the Resovia nuts from August.

PCA explained 83.10% of the overall diversity, with the first and second factors explaining 61.89% and 21.21% of the intergroup variation (Figure 1b), respectively. Figure 1b shows the projection of variables on the plane of factors. Strong negative correlations between the values of factor 1 and the parameters describing the content of individual tocopherol fractions are clearly visible. It probably results from a strong, statistically significant positive correlation between these values (*r* = 0.578–0.891). At the same time, these values do not play a major role in the factor 2 values. A similar situation occurs between the values of the antioxidative potential determined by ABTS, DPPH and TPC and factor 1. Here also a strong positive correlation between the antioxidant capacity values (*r* = 0.810–0.841) is noticed. The FRAP parameter influences the factor 2 value to the greatest extent. In addition, the almost perpendicular positioning of the FRAP parameter to the dry matter and fat content indicates a lack of correlation between them.

Figure 1c shows the projection of samples of various nut varieties with different degrees of maturity on the plane of factors. The analysis of the relationship between factor 1 and 2 points to significant similarities between the nuts of Apollo and Leopold cultivars obtained in August and September, respectively. The nuts Leopold VII and Resovia IX are clearly distinguishable from the rest.

## 4. Conclusions

Walnuts are beneficial tree nuts with great potential for the human healthspan and lifespan. They are characterized by a very high fat content, which consists of the necessary unsaturated fatty acids. In addition, they show significant antioxidant potential. Nevertheless, as shown in this study, the content of valuable bioactive substances (polyphenols, tocopherols) in walnuts significantly depends not only on their variety, but also on the degree of maturity. It was found that both, the antioxidant potential and the polyphenol content associated with it, decreased during the maturation of nuts. Unripe nuts are rich in ellagitannin, with strong antioxidant properties. In the polyphenol profile of nuts, 26 compounds that enhance their antioxidant properties have been identified. However, the content of tocopherols in nut oil increased as they matured. The high antioxidant potential and the content of bioactive compounds of unripe walnuts may contribute to the design of food products with pro-health qualities based on these raw materials.

## Figures and Tables

**Table 1:** Parameters showing physicochemical properties of walnuts in different stages of ripeness.

Cultivar	Dry Mass Content [%]	Fat Content [g/100g d.m.]	Ash Content [mg/100g d.m.]
	**July**		
**Apollo**	24.28^b^ ± 0.78	5.23^a^ ± 0.48	5.92^ef^ ± 0.58
**Leopold**	23.52^b^ ± 0.38	5.99^ab^ ± 0.23	5.46^de^ ± 0.28
**Resovia**	20.86^a^ ± 0.67	6.78^b^ ± 0.76	6.45^f^ ± 0.34
	**August**		
**Apollo**	33.54^d^ ± 0.79	11.18^c^ ± 0.54	4.71^c^ ± 0.14
**Leopold**	31.63^c^ ± 1.12	13.23^d^ ± 1.19	4.39^bc^ ± 0.29
**Resovia**	30.54^c^ ± 1.12	13.39^d^ ± 0.63	5.33^d^ ± 0.30
	**September**		
**Apollo**	64.64^f^ ± 0.83	23.47^f^ ± 0.82	3.76^a^ ± 0.13
**Leopold**	61.93^e^ ± 0.81	20.21^e^ ± 0.93	3.83^ab^ ± 0.33
**Resovia**	67.95^g^ ± 0.50	24.25^f^ ± 0.59	3.86^ab^ ± 0.24
	**Two-factor ANOVA-*p***		
**Factor 1**	<0.001	<0.001	<0.001
**Factor 2**	<0.001	<0.001	<0.001
**Factor 1 × factor 2**	<0.001	<0.001	0.123

Mean values of three replicates marked with the same letter in the column do not differ significantly at a significance level of 0.05. Factor 1—harvest time (degree of nut ripeness). Factor 2—cultivar of nuts. Factor 1 × factor 2—interaction between harvest time and the cultivar of nuts. ± standard deviation.

**Table 2:** Antioxidative potential of walnuts of different cultivars, in various stages of ripeness.

Cultivar	ABTS	DPPH	FRAP	TotalPolyphenols
	[mmol TE/100g d.m.]			[mg GAE/100g d.m.]
		**July**			
**Apollo**	52.75^e^ ± 4.22	47.14^f^ ± 5.23	96.75^f^ ± 1.20	1066.38^f^ ± 25.40
**Leopold**	82.75^f^ ± 2.09	73.54^h^ ± 0.34	107.55^g^ ± 0.90	2149.08^g^ ± 31.52
**Resovia**	11.63^b^ ± 0.91	28.82^bc^ ± 0.77	85.95^c^ ± 1.20	715.86^c^ ± 15.69
	**August**			
**Apollo**	54.90^e^ ± 0.52	40.49^de^ ± 2.18	93.15^e^ ± 1.20	937.24^e^ ± 33.86
**Leopold**	41.70^d^ ± 1.56	57.07^g^ ± 5.24	82.35^c^ ± 1.20	772.27^d^ ± 18.79
**Resovia**	3.37^a^ ± 0.64	25.87^ab^ ± 4.02	103.95^h^ ± 2.51	568.57^b^ ± 20.77
	**September**			
**Apollo**	2.68^a^ ± 0.75	38.53^de^ ± 1.86	89.55^d^ ± 1.20	964.12^e^ ± 42.59
**Leopold**	16.83^c^ ± 1.84	36.67^cd^ ± 1.29	78.75^b^ ± 1.20	688.42^c^ ± 35.51
**Resovia**	1.91^a^ ± 1.22	19.49^a^ ± 0.76	50.35^a^ ± 5.23	498.12^a^ ± 8.05
	**Two-factor ANOVA-*p***			
**Factor 1**	<0.001	<0.001	<0.001	<0.001
**Factor 2**	<0.001	<0.001	<0.001	<0.001
**Factor 1 × factor 2**	<0.001	<0.001	<0.001	<0.001

Mean values of three replicates marked with the same letter in the column do not differ significantly at the significance level of 0.05. Factor 1—harvest time (degree of nut ripeness). Factor 2—cultivar of nuts. Factor 1 × factor 2—interactions between the harvest time nuts and the cultivar of nuts. ± standard deviation.

**Table 3:** Individual phenolic compounds identified by ultraperformance liquid chromatography (UPLC).

Compound		Rt	[M − H] *m*/*z*	
		min	MS	MS/MS
1	Quinic acid	1.11	191	85, 111
2	Gallic acid	1.39	169	125
3	Pedunculagin/casuariin isomer (bis-HHDP-glucose)	1.86	783	481, 301
4	Praecoxin A/platycariin isomer (trigalloyl-HHDP-glucose)	2.30	951	907, 783, 481, 301
5	Procyanidin tetramer	2.68	576[M − H] ^2-^	865, 576, 289
6	Pedunculagin/casuarrin isomer (bis-HHDP-glucose)	2.68	783	481, 300, 275
7	Coumarylquinic acid	2.81	337	163, 119
8	Casuarinin/casuarictin isomer	3.16	935	783, 481, 301
9	Reginin A/reginin D isomer	3.35	935	783, 481, 301
10	Glansirin C isomer	3.52	933	631, 481, 301
11	Ellagic acid hexoside	3.53	463	301
12	Glansirin C isomer	3.54	933	631, 451, 301
13	Casuarinin/casuarictin isomer	3.67	935	783, 481, 301
14	Casuarinin/casuarictin isomer	3.77	935	783, 481, 301
15	Glansirin D/degalloyl rugosin F isomer	3.77	859[M − H] ^2-^	1095, 935, 633, 301
16	Casuarinin/casuarictin isomer	4.12	935	783, 481, 301
17	Praecoxin A methyl ester	4.22	965	783, 481, 301
18	Tetragalloyl-glucose	4.54	787	635, 465, 169
19	Eucalbanin A/cornusiin B isomer	4.69	1085	783, 633, 301
20	2,7-dimethyl-2,4-diene-deca-α.ω-diacid-8-*O*-glucoside	4.89	403	223, 161
21	Glansirin D/degalloyl rugosin F isomer	5.10	859[M − H] ^2-^	1095, 935, 633, 301
22	Glansirin C isomer	5.17	933	631, 481, 301
23	Heterophylliin D	5.35	934[M − H] ^2-^	1085, 783, 633, 301
24	Strictinin/isostrictinin isomer (galloyl-HHDP-glucose)	6.01	633	463, 301
25	Glansirin B isomer	6.29	905	763, 481, 301
26	Eucalbanin A/cornusiin B isomer	6.79	1085	783, 633, 451, 301

**Table 4:** Quantitative composition of polyphenols identified in walnuts (μg/g).

Compound		Rt	[M − H] *m*/*z*	Cultivar								
		min	MS	Apollo	Leopold	Resovia	Apollo	Leopold	Resovia	Apollo	Leopold	Resovia
				July			August			September		
**1**	Quinic acid	1.11	191	7.34	10.40	5.04	8.89	7.32	5.21	13.95	4.94	5.29
**2**	Gallic acid	1.39	169	31.29	33.78	11.17	35.32	17.84	16.33	32.18	11.97	10.33
**3**	Pedunculagin/casuariin isomer (bis-HHDP-glucose)	1.86	783	18.04	28.11	3.30	13.78	10.54	3.42	15.53	3.47	4.84
**4**	Praecoxin A/platycariin isomer (trigalloyl-HHDP-glucose)	2.30	951	64.98	62.45	58.07	45.83	54.93	32.58	41.78	26.30	22.85
**5**	Procyanidin tetramer	2.68	576[M − H] ^2-^	3.25	18.63	2.22	3.19	2.27	2.92	5.37	2.30	2.82
**6**	Pedunculagin/casuarrin isomer (bis-HHDP-glucose)	2.68	783	2.33	8.52	2.06	4.39	1.44	1.58	4.71	1.84	2.22
**7**	Coumarylquinic acid	2.81	337	33.40	121.59	13.73	36.28	16.95	9.48	49.46	22.58	11.20
**8**	Casuarinin/casuarictin isomer	3.16	935	4.67	14.14	3.43	6.45	3.71	2.78	9.29	3.05	2.93
**9**	Reginin A/reginin D isomer	3.35	935	8.29	16.60	4.77	4.74	6.30	2.81	7.28	3.94	2.95
**10**	Glansirin C isomer	3.52	933	7.49	36.40	2.19	4.46	1.66	2.85	6.18	2.34	3.21
**11**	Ellagic acid hexoside	3.53	463	10.56	23.69	8.17	13.49	10.16	7.76	14.16	7.74	5.65
**12**	Glansirin C isomer	3.54	933	3.19	3.81	1.39	3.13	1.47	2.05	3.54	1.84	1.99
**13**	Casuarinin/casuarictin isomer	3.67	935	6.94	39.07	3.24	4.46	2.60	3.09	3.63	2.47	2.96
**14**	Casuarinin/casuarictin isomer	3.77	935	4.88	13.40	2.95	11.58	5.41	4.39	10.90	5.00	3.41
**15**	Glansirin D/degalloyl rugosin F isomer	3.77	859[M − H] ^2-^	4.27	8.22	2.75	4.81	2.67	5.27	6.16	5.08	6.41
**16**	Casuarinin/casuarictin isomer	4.12	935	2.62	2.43	1.57	4.43	1.43	1.15	46.28	1.60	1.11
**17**	Praecoxin A methyl ester	4.22	965	3.51	7.98	3.25	5.85	1.38	3.09	5.36	4.36	2.55
**18**	Tetragalloyl-glucose	4.54	787	14.15	27.56	14.33	22.90	14.29	8.93	20.74	9.14	5.64
**19**	Eucalbanin A/cornusiin B isomer	4.69	1085	14.90	16.42	7.29	13.88	7.84	4.09	15.73	4.69	4.54
**20**	2,7-dimethyl-2,4-diene-deca-α.ω-diacid-8-*O*-glucoside	4.89	403	3.76	11.95	10.13	5.13	4.46	9.14	4.29	3.23	2.02
**21**	Glansirin D/degalloyl rugosin F isomer	5.10	859[M − H] ^2-^	2.93	14.98	1.71	5.48	3.38	1.47	4.36	2.65	1.60
**22**	Glansirin C isomer	5.17	933	2.32	4.21	2.32	2.87	2.20	1.96	3.08	1.68	1.72
**23**	Heterophylliin D	5.35	934[M − H] ^2-^	4.23	8.62	1.30	4.45	6.95	1.24	3.67	1.53	1.16
**24**	Strictinin/isostrictinin isomer (galloyl-HHDP-glucose)	6.01	633	2.93	2.43	1.57	1.06	1.56	1.40	1.78	1.44	1.52
**25**	Glansirin B isomer	6.29	905	3.39	5.07	3.46	2.04	2.76	2.16	2.43	2.23	1.84
**26**	Eucalbanin A/cornusiin B isomer	6.79	1085	11.16	2.96	2.59	6.15	8.33	2.61	3.49	4.15	2.00
TOTAL				276.81	543.40	174.01	275.04	199.90	139.76	335.34	141.56	114.77

**Table 5:** Content of tocopherols in walnuts of different cultivars, in different stages of ripeness.

Cultivar	α-Tocopherol		γ-Tocopherol		Sum β and δ-Tocopherol		Total
	mg/100g d.m.	%	mg/100g d.m.	%	mg/100g d.m.	%	mg/100g d.m.
	**July**						
**Apollo**	0.00^a^ ± 0.00	0	1.76^a^ ± 0.02	100	0.00^a^ ± 0.00	0	1.76^a^ ± 0.02
**Leopold**	0.47^b^ ± 0.33	21	1.46^a^ ± 0.06	65	0.32^b^ ± 0.02	14	2.25^a^ ± 0.11
**Resovia**	3.08^g^ ± 0.21	45	2.73^b^ ± 0.20	40	1.00^c^ ± 0.16	15	6.81^c^ ± 0.25
	**August**						
**Apollo**	2.17^e^ ± 0.05	22	3.35^c^ ± 0.05	36	3.91^e^ ± 0.25	42	9.38 ± 0.25
**Leopold**	1.56^d^ ± 0.03	44	1.56^a^ ± 0.20	44	0.43^b^ ± 0.07	12	3.55^b^ ± 0.28
**Resovia**	3.70 ± 0.01	29	3.53^c^ ± 0.03	27	5.73^g^ ± 0.06	44	12.97^e^ ± 0.10
	**September**						
**Apollo**	7.77^h^ ± 0.12	44	5.30^d^ ± 0.02	30	4.70^f^ ± 0.03	26	17.73^f^ ± 0.08
**Leopold**	1.06^c^ ± 0.10	13	5.03^d^ ± 0.47	61	2.15^d^ ± 0.22	26	8.25^d^ ± 0.78
**Resovia**	2.88^f^ ± 0.12	16	5.87^e^ ± 0.06	28	10.37^h^ ± 0.03	56	18.30^g^ ± 0.01
	**Two-factor ANOVA-*p***						
**factor 1**	<0.001		<0.001		<0.001		<0.001
**factor 2**	<0.001		<0.001		<0.001		<0.001
**factor 1 × factor 2**	<0.001		<0.001		<0.001		<0.001

Mean values of three replicates marked with the same letter in the column do not differ significantly at the significance level of 0.05.Factor 1—harvest time (degree of nut ripeness).Factor 2—cultivar of nuts.Factor 1 × factor 2—interactions between harvest time and cultivar of nuts. ± standard deviation.
